# Maximizing Fit for Cloth and Medical Procedure Masks to Improve Performance and Reduce SARS-CoV-2 Transmission and Exposure, 2021

**DOI:** 10.15585/mmwr.mm7007e1

**Published:** 2021-02-19

**Authors:** John T. Brooks, Donald H. Beezhold, John D. Noti, Jayme P. Coyle, Raymond C. Derk, Francoise M. Blachere, William G. Lindsley

**Affiliations:** ^1^CDC COVID-19 Emergency Response Team. ^2^Health Effects Laboratory Division, National Institute for Occupational Safety and Health, CDC.

Universal masking is one of the prevention strategies recommended by CDC to slow the spread of SARS-CoV-2, the virus that causes coronavirus disease 2019 (COVID-19) (*1*). As of February 1, 2021, 38 states and the District of Columbia had universal masking mandates. Mask wearing has also been mandated by executive order for federal property* as well as on domestic and international transportation conveyances.^†^ Masks substantially reduce exhaled respiratory droplets and aerosols from infected wearers and reduce exposure of uninfected wearers to these particles. Cloth masks^§^ and medical procedure masks^¶^ fit more loosely than do respirators (e.g., N95 facepieces). The effectiveness of cloth and medical procedure masks can be improved by ensuring that they are well fitted to the contours of the face to prevent leakage of air around the masks’ edges. During January 2021, CDC conducted experimental simulations using pliable elastomeric source and receiver headforms to assess the extent to which two modifications to medical procedure masks, 1) wearing a cloth mask over a medical procedure mask (double masking) and 2) knotting the ear loops of a medical procedure mask where they attach to the mask’s edges and then tucking in and flattening the extra material close to the face (knotted and tucked masks), could improve the fit of these masks and reduce the receiver’s exposure to an aerosol of simulated respiratory droplet particles of the size considered most important for transmitting SARS-CoV-2. The receiver’s exposure was maximally reduced (>95%) when the source and receiver were fitted with modified medical procedure masks. These laboratory-based experiments highlight the importance of good fit to optimize mask performance. Until vaccine-induced population immunity is achieved, universal masking is a highly effective means to slow the spread of SARS-CoV-2** when combined with other protective measures, such as physical distancing, avoiding crowds and poorly ventilated indoor spaces, and good hand hygiene. Innovative efforts to improve the fit of cloth and medical procedure masks to enhance their performance merit attention.

At least two recent studies examined use of mask fitters to improve the fit of cloth and medical procedure masks. Fitters can be solid (*2*) or elastic (*3*) and are worn over the mask, secured with head ties or ear loops. The results indicated that when fitters are secured over a medical procedure mask, they can potentially increase the wearer’s protection by ≥90% for aerosols in the size range considered to be the most important for transmitting SARS-CoV-2 (generally <10 *μ*m). Other studies found that knotting and tucking a medical procedure mask or placing a sleeve made of sheer nylon hosiery material around the neck and pulling it up over either a cloth or medical procedure mask (*3**,**4*) also significantly improved the wearer’s protection by fitting the mask more tightly to the wearer’s face and reducing edge gaps. A recent expert commentary (*5*) proposed double masking as another means to improve the fit of medical procedure masks and maximize the filtration properties of the materials from which they are typically constructed, such as spun-bond and melt-blown polypropylene. Based on experiments that measured the filtration efficiencies of various cloth masks and a medical procedure mask (*6*), it was estimated that the better fit achieved by combining these two mask types, specifically a cloth mask over a medical procedure mask, could reduce a wearer’s exposure by >90%.

During January 2021, CDC conducted various experiments to assess two methods to improve medical procedure mask performance by improving fit and, in turn, filtration: 1) double masking and 2) knotting and tucking the medical procedure mask (Figure 1). The first experiment assessed how effectively various mask combinations reduced the amount of particles emitted during a cough (i.e., source control) in terms of collection efficiency. A pliable elastomeric headform was used to simulate a person coughing by producing aerosols from a mouthpiece (0.1–7 *μ*m potassium chloride particles) (*7*). The effectiveness of the following mask configurations to block these aerosols was assessed: a three-ply medical procedure mask alone, a three-ply cloth cotton mask alone, and the three-ply cloth mask covering the three-ply medical procedure mask (double masking). The second experiment assessed how effectively the two modifications to medical procedure masks reduced exposure to aerosols emitted during a period of breathing. Ten mask combinations, using various configurations of no mask, double masks, and unknotted or knotted and tucked medical procedure masks, were assessed (e.g., source with no mask and receiver with double mask or source with double mask and receiver with no mask). A knotted and tucked medical procedure mask is created by bringing together the corners and ear loops on each side, knotting the ears loops together where they attach to the mask, and then tucking in and flattening the resulting extra mask material to minimize the side gaps^††^ (Figure 1). A modified simulator with two pliable elastomeric headforms (a source and a receiver) was used to simulate the receiver’s exposure to aerosols produced by the source (*8*). In a chamber approximately 10 ft (3.1 m) long by 10 ft wide by 7 ft (2.1 m) high, which simulated quiet breathing during moderate work, the source headform was programmed to generate the aerosol from its mouthpiece at 15 L/min (International Organization for Standardization [ISO] standard for a female performing light work), and the receiver headform’s minute ventilation was set at 27 L/min (ISO average of a male or female engaged in moderate work).^§§^ For each of the 10 masking configurations, three 15-minute runs were completed. 

**FIGURE 1:**
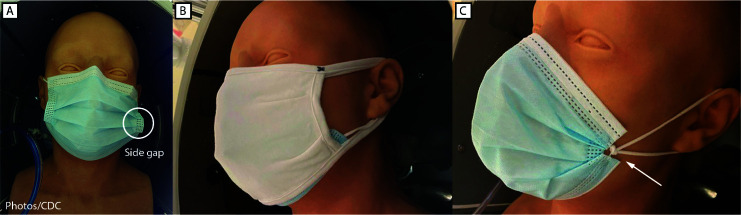
Masks tested, including *A,* unknotted medical procedure mask; *B,* double mask (cloth mask covering medical procedure mask); and *C,* knotted/tucked medical procedure mask.

Results from the first experiment demonstrated that the unknotted medical procedure mask alone blocked 56.1% of the particles from a simulated cough (standard deviation [SD] = 5.8), and the cloth mask alone blocked 51.4% (SD = 7.1). The combination of the cloth mask covering the medical procedure mask (double mask) blocked 85.4% of the cough particles (SD = 2.4), and the knotted and tucked medical procedure mask blocked 77.0% (SD = 3.1).

In the second experiment, adding a cloth mask over the source headform’s medical procedure mask or knotting and tucking the medical procedure mask reduced the cumulative exposure of the unmasked receiver by 82.2% (SD = 0.16) and 62.9% (SD = 0.08), respectively (Figure 2). When the source was unmasked and the receiver was fitted with the double mask or the knotted and tucked medical procedure mask, the receiver’s cumulative exposure was reduced by 83.0% (SD = 0.15) and 64.5% (SD = 0.03), respectively. When the source and receiver were both fitted with double masks or knotted and tucked masks, the cumulative exposure of the receiver was reduced 96.4% (SD = 0.02) and 95.9% (SD = 0.02), respectively. 

**FIGURE 2:**
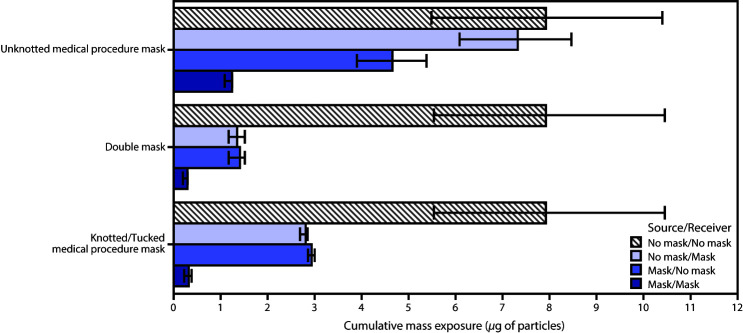
Mean cumulative exposure* for various combinations of no mask, double masks, and unknotted and knotted/tucked medical procedure masks† * To an aerosol of 0.1–7 *μ*m potassium chloride particles (with 95% confidence intervals indicated by error bars) measured at mouthpiece of receiver headform configured face to face 6 ft from a source headform, with no ventilation and replicated 3 times. Mean improvements in cumulative exposures compared with no mask/no mask (i.e., no mask wearing, or 100% exposure) were as follows: *unknotted medical procedure mask*: no mask/mask = 7.5%, mask/no mask = 41.3%, mask/mask = 84.3%; *double mask*: no mask/mask = 83.0%, mask/no mask = 82.2%, mask/mask = 96.4%; *knotted/tucked medical procedure mask*: no mask/mask = 64.5%, mask/no mask = 62.9%, mask/mask = 95.9%. ^†^ Double mask refers to a three-ply medical procedure mask covered by a three-ply cloth cotton mask. A knotted and tucked medical procedure mask is created by bringing together the corners and ear loops on each side, knotting the ears loops together where they attach to the mask, and then tucking in and flattening the resulting extra mask material to minimize the side gaps.

## Discussion

These laboratory-based experiments highlight the importance of good fit to maximize overall mask performance. Medical procedure masks are intended to provide source control (e.g., maintain the sterility of a surgical field) and to block splashes. The extent to which they reduce exhalation and inhalation of particles in the aerosol size range varies substantially, in part because air can leak around their edges, especially through the side gaps (*9*). The reduction in simulated inhalational exposure observed for the medical procedure mask in this report was lower than reductions reported in studies of other medical procedure masks that were assessed under similar experimental conditions, likely because of substantial air leakage around the edges of the mask used here (*10*). In another study, adding mask fitters to two medical procedure masks, which produced different reductions in exposure when unmodified, enhanced their efficiencies to the same equally high levels (*2*). This observation suggests that modifications to improve fit might result in equivalent improvements, regardless of the masks’ baseline filtration efficiencies.

The findings in this report are subject to at least four limitations. First, these experiments were conducted with one type of medical procedure mask and one type of cloth mask among the many choices that are commercially available and were intended to provide data about their relative performance in a controlled setting. The findings of these simulations should neither be generalized to the effectiveness of all medical procedure masks or cloths masks nor interpreted as being representative of the effectiveness of these masks when worn in real-world settings. Second, these experiments did not include any other combinations of masks, such as cloth over cloth, medical procedure mask over medical procedure mask, or medical procedure mask over cloth. Third, these findings might not be generalizable to children because of their smaller size or to men with beards and other facial hair, which interfere with fit. Finally, although use of double masking or knotting and tucking are two of many options that can optimize fit and enhance mask performance for source control and for wearer protection, double masking might impede breathing or obstruct peripheral vision for some wearers, and knotting and tucking can change the shape of the mask such that it no longer covers fully both the nose and the mouth of persons with larger faces. 

Controlling SARS-CoV-2 transmission is critical not only to reduce the widespread effects of the COVID-19 pandemic on human health and the economy but also to slow viral evolution and the emergence of variants that could alter transmission dynamics or affect the usefulness of diagnostics, therapeutics, and vaccines. Until vaccine-induced population immunity is achieved, universal masking is a highly effective means to slow the spread of SARS-CoV-2 when combined with other protective measures, such as physical distancing, avoiding crowds and poorly ventilated indoor spaces, and good hand hygiene. The data in this report underscore the finding that good fit can increase overall mask efficiency. Multiple simple ways to improve fit have been demonstrated to be effective. Continued innovative efforts to improve the fit of cloth and medical procedure masks to enhance their performance merit attention.

**Summary**

**What is already known about this topic?**

Universal masking is recommended to slow the spread of COVID-19. Cloth masks and medical procedure masks substantially reduce exposure from infected wearers (source control) and reduce exposure of uninfected wearers (wearer exposure).

**What is added by this report?**

CDC conducted experiments to assess two ways of improving the fit of medical procedure masks: fitting a cloth mask over a medical procedure mask, and knotting the ear loops of a medical procedure mask and then tucking in and flattening the extra material close to the face. Each modification substantially improved source control and reduced wearer exposure.

**What are the implications for public health?**

These experiments highlight the importance of good fit to maximize mask performance. There are multiple simple ways to achieve better fit of masks to more effectively slow the spread of COVID-19.

## Footnotes

* https://www.whitehouse.gov/briefing-room/presidential-actions/2021/01/20/executive-order-protecting-the-federal-workforce-and-requiring-mask-wearing

† https://www.whitehouse.gov/briefing-room/presidential-actions/2021/01/21/executive-order-promoting-covid-19-safety-in-domestic-and-international-travel

§ A cloth mask refers to any mask constructed from textiles or fabrics (both natural and synthetic) that is not a surgical mask or N95 respirator and is not intended for use as personal protective equipment. At present, there are no national standards established for cloth masks although such standards are under consideration by ASTM (formerly known as American Society for Testing and Materials).

¶ A medical procedure mask refers to any commercially produced mask regulated by the Food and Drug Administration under 21 CFR 878.4040 for performing medical procedures. These are variably labeled as surgical, laser, isolation, dental, or medical procedure masks. They may be variably shaped, including flat pleated, cone shaped, or duck bill. Medical procedure masks are loose fitting and are not expected to provide a reliable level of protection against airborne or aerosolized particles as N95 respirators regulated by the National Institute for Occupational Safety and Health. A more detailed comparison of medical procedure masks and respirators is available. https://www.cdc.gov/niosh/npptl/pdfs/UnderstandDifferenceInfographic-508.pdf

**
https://www.cdc.gov/coronavirus/2019-ncov/more/masking-science-sars-cov2.html

†† https://youtu.be/UANi8Cc71A0

§§
https://www.iso.org/standard/67530.html
